# Outcome of 1051 Octogenarian Patients With ST‐Segment Elevation Myocardial Infarction Treated With Primary Percutaneous Coronary Intervention: Observational Cohort From the London Heart Attack Group

**DOI:** 10.1161/JAHA.115.003027

**Published:** 2016-06-27

**Authors:** Daniel I. Bromage, Daniel A. Jones, Krishnaraj S. Rathod, Claire Grout, M. Bilal Iqbal, Pitt Lim, Ajay Jain, Sundeep S. Kalra, Tom Crake, Zoe Astroulakis, Mick Ozkor, Roby D. Rakhit, Charles J. Knight, Miles C. Dalby, Iqbal S. Malik, Anthony Mathur, Simon Redwood, Philip A. MacCarthy, Andrew Wragg

**Affiliations:** ^1^ Barts Health NHS TrustSt Bartholomew's HospitalLondonUK; ^2^ Royal Brompton & Harefield NHS Foundation TrustHarefield HospitalMiddlesexUK; ^3^ St. George's Healthcare NHS Foundation TrustSt. George's HospitalLondonUK; ^4^ UCL Hospitals NHS Foundation TrustHeart HospitalLondonUK; ^5^ Kings College HospitalKing's College Hospital NHS Foundation TrustLondonUK; ^6^ Royal Free London NHS Foundation TrustLondonUK; ^7^ Imperial College Healthcare NHS Foundation TrustHammersmith HospitalLondonUK; ^8^ BHF Centre of ExcellenceKing's College LondonSt. Thomas HospitalLondonUK; ^9^ The Hatter Cardiovascular InstituteUniversity College LondonLondonUK

**Keywords:** acute myocardial infarction, aging, cardiovascular disease, complications, elderly, epidemiology, octogenarian, outcome, primary percutaneous coronary intervention, Coronary Artery Disease, Aging, Myocardial Infarction, Stent

## Abstract

**Background:**

ST‐segment elevation myocardial infarction is increasingly common in octogenarians, and optimal management in this cohort is uncertain. This study aimed to describe the outcomes of octogenarians with ST‐segment elevation myocardial infarction treated by primary percutaneous coronary intervention.

**Methods and Results:**

We analyzed 10 249 consecutive patients with ST‐segment elevation myocardial infarction treated with primary percutaneous coronary intervention between 2005 and 2011 at 8 tertiary cardiac centers across London, United Kingdom. The primary end point was all‐cause mortality at a median follow‐up of 3 years. In total, 1051 patients (10.3%) were octogenarians, with an average age of 84.2 years, and the proportion increased over the study period (*P*=0.04). In‐hospital mortality (7.7% vs 2.4%, *P*<0.0001) and long‐term mortality (51.6% vs 12.8%, *P*<0.0001) were increased in octogenarians compared with patients aged <80 years, and age was an independent predictor of mortality in a fully adjusted model (hazard ratio 1.07, 95% CI 1.07–1.09, *P*<0.0001). Time‐stratified analysis revealed an increasingly elderly and more complex cohort over time. Nonetheless, long‐term mortality rates among octogenarians remained static over time, and this may be attributable to improved percutaneous coronary intervention techniques, including significantly higher rates of radial access and lower bleeding complications. Variables associated with bleeding complications were similar between octogenarian and younger cohorts.

**Conclusions:**

In this large registry, octogenarians undergoing primary percutaneous coronary intervention had a higher rate of complications and mortality compared with a younger population. Over time, octogenarians undergoing primary percutaneous coronary intervention increased in number, age, and complexity. Nevertheless, in‐hospital outcomes were reasonable, and long‐term mortality rates were static.

## Introduction

Western populations are gradually aging. Between 1985 and 2010, the number of people aged >80 years in the developed world has more than doubled to 53 million, and this trend is predicted to continue.^1^ Coronary artery disease is common in elderly patients: An estimated 13% of ST‐segment elevation myocardial infarction (STEMI) in Western cohorts occurs in patients aged >80 years.^2^ Consequently, as the population ages, we can expect to see more elderly patients presenting with STEMI. Furthermore, STEMI in elderly patients is associated with high mortality, estimated to be up to 47% at 1 year, which is unsurprising, given that comorbidities increase with age.^3^, ^4^, ^5^, ^6^, ^7^, ^8^

Primary percutaneous coronary intervention (PPCI) has been shown to be beneficial in younger patients with STEMI and is increasingly being used in octogenarians.^9^ PPCI in octogenarians is associated with improved survival compared with medical therapy alone, albeit often in subgroup analyses;^10^, ^11^, ^12^, ^13^, ^14^, ^15^ however, advanced age has been associated with worse outcomes after PPCI compared with younger patients in several recent cohort studies.^2^, ^16^, ^17^, ^18^, ^19^ Moreover, there is a paucity of evidence on the optimal management of STEMI in octogenarians as a result of their underrepresentation in clinical trials, the perceived increased risks related to comorbidities, and the presumed limited scope for symptomatic improvement. Nonetheless, there is no upper age limit for offering PPCI in current guidelines.^20^, ^21^

In this large cohort of elderly patients, we aimed to describe the population and their short‐ and long‐term outcomes after PPCI for STEMI. We also compared the clinical outcomes with a younger group of patients, examined changes over time, and investigated any associations between outcome and procedural characteristics.

## Methods

This retrospective observational cohort study investigated the characteristics and outcomes of octogenarians after PPCI for STEMI compared with nonoctogenarian patients. We merged the databases of the 8 heart attack centers in London, United Kingdom, that collect data based on the British Cardiac Intervention Society (BCIS) data set. The BCIS audit is part of a national mandatory audit in which all UK PCI centers participate.

### BCIS–National Institute for Cardiovascular Outcomes Research Database

The UK BCIS audit collects data from all hospitals in the United Kingdom that perform PCI and records information about every procedure performed.^22^ PCI is defined as the use of any coronary device to approach, probe, or cross ≥1 coronary lesion, with the intention of performing a coronary intervention. The database is part of the suite of data sets collected under the auspices of the National Institute for Cardiovascular Outcomes Research and is compliant with UK data protection legislation. Data are collected prospectively at each hospital, encrypted electronically, and transferred online to a central database. Each patient entry offers details of the patient journey, including the method and timing of admission, inpatient investigations, results, treatment, and outcomes. Patient survival data are obtained by linkage of patient National Health Service (NHS) numbers to the Office of National Statistics, which records live status and the date of death for all deceased patients.

### Population Study and Design

We examined an observational cohort of consecutive patients with STEMI treated with PPCI between January 2005 and July 2011 at all 8 tertiary cardiac centers in London. Patient and procedural details were recorded at the time of the procedure and during the admission into each center's local BCIS database. Anonymous data sets with linked mortality data from the Office of National Statistics from the 8 centers were merged for analysis. During the study period, 11 466 patients underwent PPCI. Of these, 10 249 (88%) had complete data sets and NHS numbers and were included in the analysis. The octogenarian subgroup included all patients aged ≥80 years at the time of PPCI, and patients did not switch groups if they passed age 80 years during the follow‐up period.

### Clinical Outcomes

Patient clinical and demographic data, procedural characteristics, bleeding complications, procedural complications, and major adverse cardiac events, including all‐cause in‐hospital mortality, nonfatal myocardial infarction (MI), reintervention, and stroke, were recorded during admission. Bleeding was defined as access‐site bleeding, gastrointestinal bleeding, intrapericardial bleeding with tamponade, or requirement for blood transfusion. Access‐site bleeding was defined as bleeding delaying discharge, large hematomas (defined as a hematoma that results in a change in management, including delay of discharge), retroperitoneal bleeding, and pseudoaneurysm formation. Major bleeding was defined as per bleeding, but only hematomas requiring blood transfusion were included. In‐hospital major adverse cardiac events were defined as death, MI (new pathological Q waves in the distribution of the treated coronary artery with an increase of creatine kinase‐MB to ≥2 times the reference value or significant rise in troponin T values), stroke, and target vessel revascularization. Procedural complications recorded included MI, emergency coronary artery bypass grafting, arterial complications, aortic or coronary dissection, side branch occlusion, and arrhythmia. Following discharge, long‐term all‐cause mortality was obtained by linkage to the Office of National Statistics (England and Wales). A successful PPCI result was defined as final TIMI (Thrombolysis In Myocardial Infarction) flow grade 3 and residual stenosis <20% in the infarct‐related artery at the end of the procedure.

### Ethics

The data were collected as part of a national cardiac audit, and all patient‐identifiable fields were removed prior to analysis. The local ethics committee advised us that formal ethics approval was not required.

### Statistical Methods

Clinical characteristics of octogenarians versus younger patients were compared using the Pearson chi‐square test for categorical variables and the Student *t* test for continuous variables. Normality of distribution was assessed using the Shapiro‐Wilks test. We calculated Kaplan–Meier product limits for cumulative probability of reaching an end point and used the log‐rank test for evidence of a statistically significant difference between the groups. Time was measured from the first admission for a procedure to outcome (all‐cause mortality). Cox regression analysis was used to estimate hazard ratios (HRs) for the effect of age in fully adjusted models, based on covariates (*P*<0.05) associated with the outcome. The proportional hazards assumption was evaluated by examining log (‐log) survival curves and tested with Schoenfield residuals and was satisfied for all outcomes evaluated. To determine independent predictors of other clinical and composite outcomes, a logistic regression model adjusting for 24 patient, clinical, and procedural variables was used to provide adjusted odds ratios (ORs) with associated 95% CIs. A *P* value <0.05 was considered significant. We used SPSS for Mac version 19.0 (IBM Corp) for all analyses.

## Results

### Patient Characteristics

**Figure 1:**
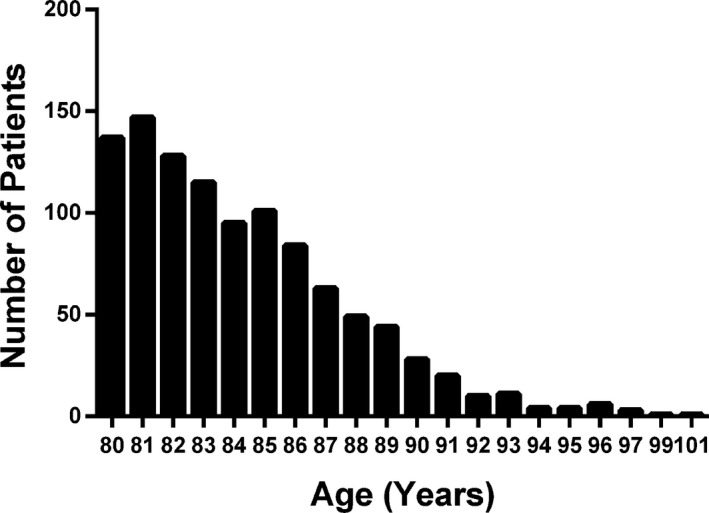
Age distribution of patients aged >80 years. The bar graph shows the absolute numbers of patients undergoing primary percutaneous coronary intervention between 2005 and 2011 according to age.

A total of 1051 octogenarians (10.3% of the study population) with an average age of 84.2 years were treated with PPCI during the study period. Over time, the annual number of octogenarians gradually increased from 47 (9.1%) in 2005 to 103 (10.5%) in 2011 (*P*=0.04). The age distribution of the study cohort is shown in Figure 1.

Compared with patients aged <80 years, octogenarian STEMI patients included a higher proportion of women and had a higher prevalence of hypertension, hypercholesterolemia, previous stroke, peripheral vascular disease, chronic renal failure, and previous coronary artery bypass grafting. They were also more likely to have worse left ventricular systolic function and to present with cardiogenic shock. The groups aged <80 years were more likely to have a smoking history and to have had previous PCI. The octogenarian group had longer call‐to‐balloon times but similar door‐to‐balloon times. Baseline characteristics are given in Table 1.

**Table 1:** Baseline Patient Characteristics According to Age

	<80 Years (n=9198)	>80 Years (n=1051)	*P* Value	Complete Data, %
Sex (female)	1800 (19.6)	474 (45.4)	<0.0001	99.8
Hypertension	3692 (42.3)	501 (51.3)	0.02	95.1
Diabetes mellitus	1398 (16.1)	137 (13.9)	0.073	94.4
Hypercholesterolemia	3708 (42.5)	548 (56.1)	<0.0001	95.1
Smoking history	5611 (61)	379 (36.1)	<0.0001	87.4
Previous MI	1442 (16.9)	182 (18.7)	0.150	92.2
Previous CABG	264 (3.0)	46 (4.6)	0.01	96.3
PVD	145 (1.7)	29 (3.0)	0.007	95.1
Previous stroke	155 (1.8)	45 (4.6)	<0.0001	95.1
Cardiogenic shock	517 (5.7)	82 (7.9)	0.004	98.5
Previous PCI	1078 (12.3)	87 (8.7)	0.001	95.6
CRF	72 (0.9)	16 (1.8)	0.017	89.7
Poor LV (<30%)	572 (15.1)	80 (20.6)	0.007	40.0
Ethnicity (white)	1081 (67.6)	697 (61.8)	0.32	91.2

Data expressed as number (%) or median (interquartile range). CABG indicates coronary artery bypass grafting; CRF, chronic renal failure; LV, left ventricle; MI, myocardial infarction; PCI, percutaneous coronary intervention; PVD, peripheral vascular disease.

### Procedural characteristics

Octogenarian patients were more likely to have multivessel disease and less likely to undergo radial access or to receive adjunctive therapies such as glycoprotein (GP) IIb/IIIa inhibitors and thrombectomy. There were lower rates of stent placement in the octogenarian group, and when stents were inserted, they were less likely to be drug‐eluting stents (DESs). The procedure was more likely to be successful in younger patients. Procedural characteristics are given in Table 2.

**Table 2:** Procedural Characteristics According to Age

	<80 Years (n=9198)	>80 Years (n=1051)	*P* Value	Complete Data, %
Radial access	2115 (23.4)	194 (18.8)	0.001	98.3
Multivessel disease	3821 (41.8)	562 (54)	<0.0001	94.6
Multivessel intervention	917 (10)	277 (26)	0.002	98.9
Target vessel in single‐vessel intervention				98.9
Right coronary artery	3352 (42.3)	392 (45.5)	0.076	
Left main coronary artery	44 (0.60)	12 (1.4)	0.010	
Left anterior descending	3458 (43.6)	360 (41.8)	0.294	
Left circumflex	1071 (13.5)	98 (11.4)	0.081	
Stent placed	8432 (91.7)	938 (89.2)	0.014	96.0
Drug‐eluting stent use	4058 (45.9)	311 (30.9)	<0.0001	96.0
Average number of stents used	1.4 (±1.13)	1.5 (±0.96)	0.569	98.4
Glycoprotein IIb/IIIa inhibitor use	6843 (74.4)	530 (50.4)	<0.0001	95.5
Thrombectomy device use	3458 (37.6)	283 (27)	<0.0001	95.5
Procedural success	6932 (88.3)	890(84.7)	0.003	84.8
Call to balloon time (min)	130 (82–171)	168 (120–220)	0.018	61.0
Door to balloon time (min)	43 (25–128)	45 (28–134)	0.562	61.0

Data expressed as number (%) or median (interquartile range).

### Procedural and In‐Hospital Complications

The rate of complications was higher in patients aged >80 years, including significantly more bleeding complications and subsequent blood transfusion. Consequently, the group aged >80 years had significantly longer in‐patient stays. In‐hospital major adverse cardiac event rates were significantly higher in the octogenarian group compared with the younger group, accounted for by significantly increased all‐cause mortality (7.7% vs 2.4%, *P*<0.0001) and Q wave MI (3.0% vs 1.7%, *P*=0.006). Procedural and in‐hospital complications are shown in Table 3.

**Table 3:** Procedural and In‐Hospital Complications

	<80 Years (n=9198)	>80 Years (n=1051)	*P* Value	Complete Data, %
Procedural complications				96.5
Total	444 (4.8)	98 (9.3)	<0.0001	
Coronary dissection	59 (0.6)	6 (0.6)	1.000	
Aortic dissection	3 (0.03)	0 (0.0)	1.000	
Coronary perforation	14 (0.2)	5 (0.5)	0.034	
Heart block	43 (0.5)	15 (1.7)	<0.0001	
No/slow flow	128 (1.4)	16 (1.8)	0.576	
Bleeding complications	87 (1.0)	36 (3.43)	0.002	
Blood Transfusion	28 (0.3)	8 (0.76)	0.026	
MACE	460 (5.0)	120 (11.4)	<0.0001	96.5
Mortality	211 (2.4)	78 (7.7)	<0.0001	
Q wave MI	149 (1.7)	30 (3.0)	0.006	
Non–Q Wave MI	26 (0.37)	3 (0.3)	1.000	
Emergency CABG	30 (0.3)	5 (0.5)	0.399	
Reintervention PCI	44 (0.48)	4 (0.38)	0.814	
Stroke	17 (0.2)	7 (0.67)	0.008	

Data expressed as number (%). CABG indicates coronary artery bypass grafting; MACE, major adverse cardiac events; MI, myocardial infarction; PCI, percutaneous coronary intervention.

### Long‐Term All‐Cause Mortality

**Figure 2:**
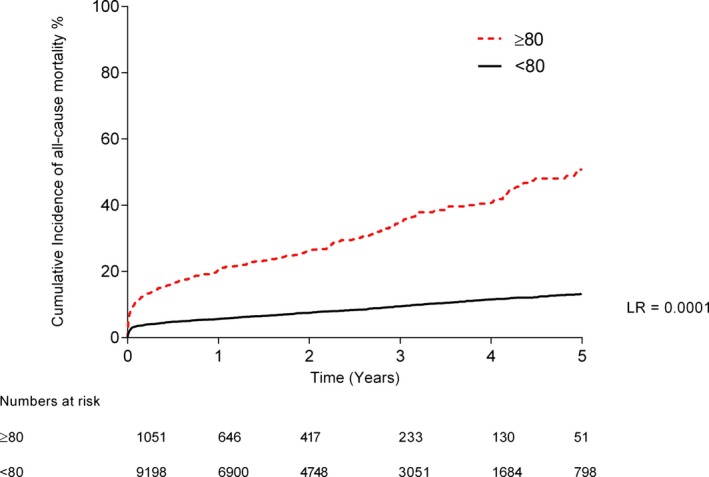
Kaplan–Meier curves showing all‐cause mortality after PPCI. Kaplan–Meier curves showing the cumulative probability of all‐cause mortality after PPCI according to group. LR indicates log‐rank; PPCI, percutaneous coronary intervention.

**Figure 3:**
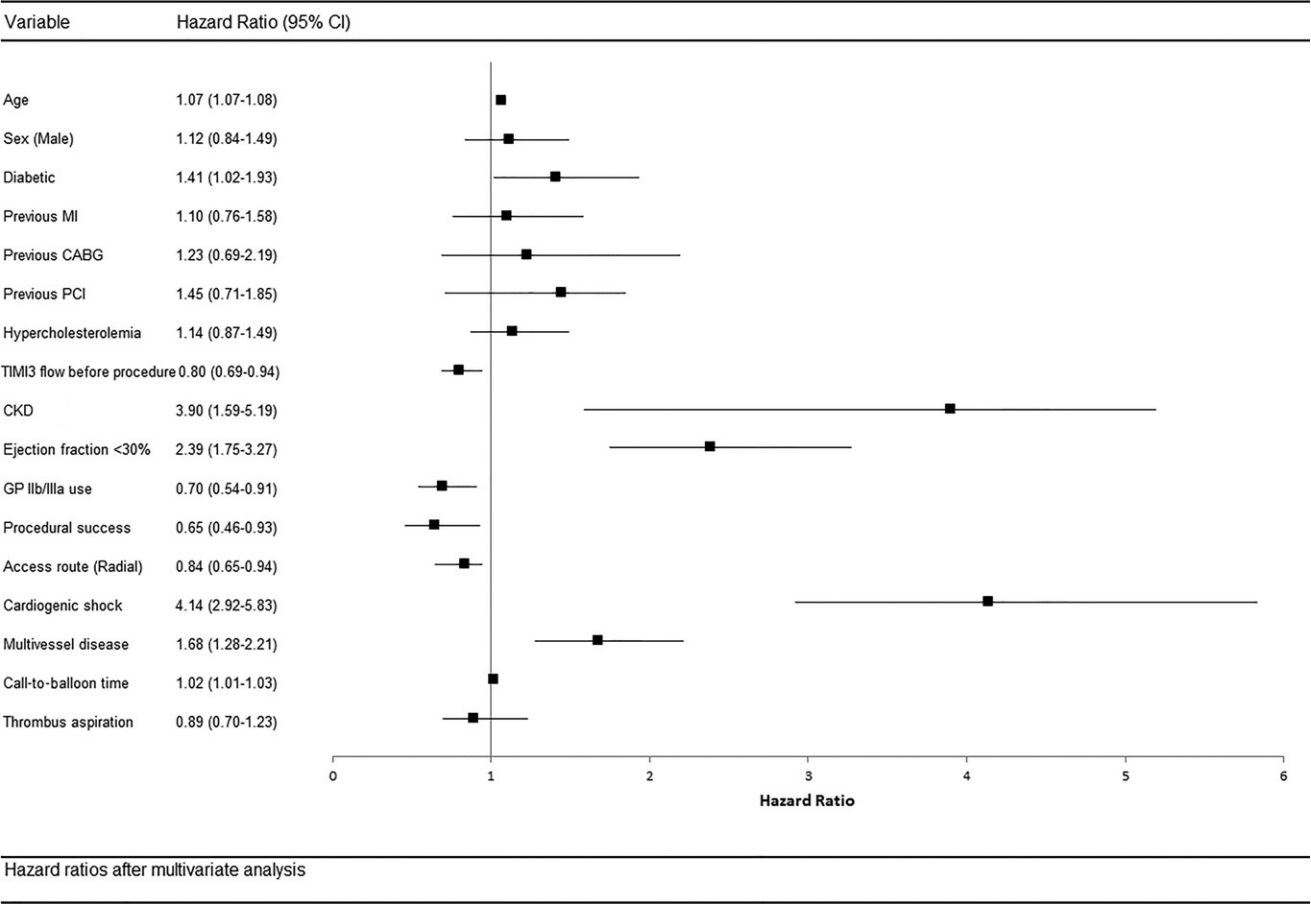
Multivariate Cox regression analysis for hazard of all‐cause mortality after PPCI. multivariate Cox regression analysis for hazard of all‐cause mortality after PPCI. CABG indicates coronary artery bypass grafting; CKD, chronic kidney disease; GP, glycoprotein; MI, myocardial infarction; PPCI, primary percutaneous coronary intervention; TIMI, Thrombolysis In Myocardial Infarction.

Kaplan–Meier analysis showed that the cumulative incidence of all‐cause mortality during follow‐up was significantly higher in the octogenarian group compared with the younger subgroup (median follow‐up 3.0 years [interquartile range 1.2–4.6 years]; 51.6% vs 12.8%, *P*<0.0001) (Figure 2). The hazard of death during follow‐up increased with age (unadjusted HR 1.07 per year increase, 95% CI 1.06–1.08, *P*<0.0001) and persisted after adjustment for other predictors of mortality (HR 1.07, 95% CI 1.07–1.09, *P*<0.0001) (Figure 3). After adjustment for confounding variables, other independent predictors of increased long‐term all‐cause mortality were cardiogenic shock, poor left ventricular function, chronic renal failure, multivessel disease, femoral access, bare metal stent use, and procedural failure.

### Bleeding Complications

Overall bleeding rates were greater in the octogenarian group (3.43% vs 1.00%, *P*=0.002%) and was driven by access‐site bleeding (1.93% vs 0.28%, *P*=0.002) and necessitated greater volume of blood transfusions (0.76% vs 0.30%, *P*=0.026). When corrected for baseline clinical and procedural variables (24‐variable model), multivariate analysis identified the following variables as independent predictors of bleeding: age (OR 1.25, 95% CI 1.10–1.42, *P*<0.0001); peripheral vascular disease (OR 3.69, 95% CI 1.20–11.37, *P*=0.023); female sex (OR 1.85, 95% CI 1.39–4.02, *P*<0.001); GP IIb/IIIa inhibitor use (OR 2.10, 95% CI 1.33–3.03, *P*=0.010); intra‐aortic balloon pump use (OR 5.45, 95% CI 1.53–15.27, *P*=0.008); and previous stroke (OR 3.49, 95% CI 1.25–8.93, *P*=0.009) with radial access (OR 0.20, 95% CI 0.10–0.77, *P*=0.002), a predictor of reduced bleeding events. Multivariate Cox analysis revealed bleeding complications to be an independent predictor of mortality (HR 2.34, 95% CI 1.34–3.56, *P*=0.002). When separated into young and octogenarian groups, no differences were seen in variables associated with bleeding complications, which matched the overall cohort (female sex, radial access, GP IIb/IIIa inhibitor use, previous stroke, peripheral vascular disease, and intra‐aortic balloon pump use).

### Octogenarian Subgroup Analysis

**Figure 4:**
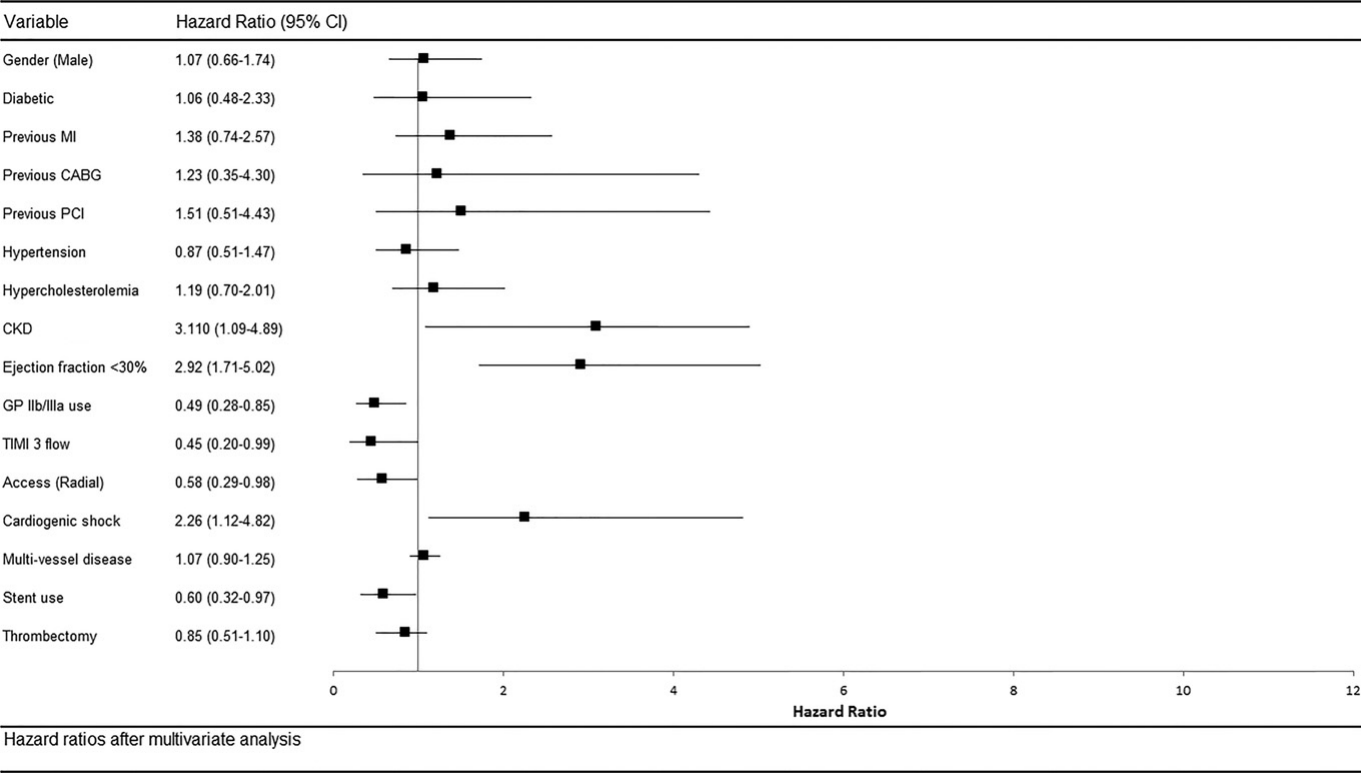
Multivariate Cox regression analysis for hazard of all‐cause mortality after PPCI in octogenarians. Multivariate Cox regression analysis for hazard of all‐cause mortality after PPCI. CABG indicates coronary artery bypass grafting; CKD, chronic kidney disease; GP, glycoprotein; MI, myocardial infarction; PPCI, primary percutaneous coronary intervention; TIMI, Thrombolysis In Myocardial Infarction.

In the octogenarian subgroup, similar univariate predictors of mortality were present, including shock, access route, and nonuse of DESs, GP IIb/IIIa, and thrombectomy (data not shown). After correction for sex and comorbidities including diabetes, previous MI, previous revascularization (coronary artery bypass grafting, PCI), hypertension, hypercholesterolemia, peripheral vascular disease, stroke, chronic renal failure, poor left ventricular function, GP IIb/IIIa use, procedural success, DES use, thrombectomy use, shock, access and multivessel disease, multivariate analysis of the octogenarian subgroup corroborated the finding in the whole study population that cardiogenic shock, femoral access, chronic renal failure, and poor left ventricular and procedural failure were independent predictors of mortality (Figure 4). In parallel to the young group, GP IIb/IIIa use (HR 0.49, 95% CI 0.28–0.85), radial access (HR 0.58, 95% CI 0.29–0.98) and DES use (HR 0.60, 95% CI 0.32–0.98) were all significantly associated with improved survival (Figure 4).

### Octogenarian Time‐Stratified Analysis

Further Kaplan–Meier analysis according to time period among the octogenarian group demonstrated that the difference in cumulative incidence of all‐cause long‐term mortality during follow‐up was not significantly different between the 2005–2008 and 2008–2011 time periods (33.8% vs 29.7%, *P*=0.89). In addition, between 2008 and 2011, octogenarian patients were significantly older. They were also more complicated, with higher rates of diabetes mellitus, previous MI, and revascularization, and significantly more likely to present with cardiogenic shock. Their procedural characteristics revealed a higher likelihood of radial access and DES use and a smaller chance of receiving either thrombectomy or a GP IIb/IIIa inhibitor (Table 4). There were higher rates of procedural success (86.4% vs 83.8%, *P*=0.031) and lower rates of bleeding complications (1.9% vs 5.4%, *P*=0.028).

**Table 4:** Time‐Stratified Analysis Among Patients Aged >80 Years Comparing 2005–2008 With 2008–2011

	2005–2008 (n=442)	2009–2011 (n=609)	*P* Value
Patient characteristics			
Age, y	83.86±3.16	84.48±3.83	<0.0001
Sex (female)	194 (43.9)	280 (46.0)	0.571
Ethnicity (white)	296 (67.1)	401 (65.8)	0.435
Cardiogenic shock	22 (5.0)	60 (9.9)	0.005
Previous MI	81 (18.3)	101 (16.6)	0.358
Previous CABG	12(2.7)	34(5.4)	0.025
Previous PCI	27 (6.1)	60 (9.9)	0.048
Smoking history	162 (36.7)	217 (35.6)	0.078
Diabetes mellitus	52 (11.8)	85 (14.0)	0.039
Stroke	21 (4.8)	24 (3.9)	0.395
Peripheral vascular disease	10 (2.3)	19 (3.1)	0.498
Procedural characteristics			
GP IIb/IIIa inhibitor use	255 (57.7)	275 (45.2)	0.005
Radial access	50 (11.3)	144 (23.6)	<0.0001
Stent placed	358 (81.1)	580 (95.2)	<0.0001
DES use	90 (20.4)	220 (36.1)	<0.0001
Average number of DESs used	0.35±0.76	0.61±0.96	<0.0001
Procedural success	370 (83.8)	520 (86.4)	0.031
Thrombectomy device use	138 (31.2)	145 (23.8)	0.032
Total bleeding complications	24 (5.4)	12 (1.9)	0.028
Major bleeding	12 (2.7)	7 (1.1)	0.082
Arterial complications	8 (1.8)	3 (0.5)	0.064
Blood transfusions	4 (0.9)	2 (0.3)	0.128

Data expressed as number (%) or mean (SD). CABG indicates coronary artery bypass grafting; DES, drug‐eluting stent; GP, glycoprotein; MI, myocardial infarction; PCI, percutaneous coronary intervention.

## Discussion

There is increasing interest in the management of octogenarian patients presenting with STEMI. This study sought to describe a population of octogenarians and their outcomes after PPCI for STEMI and to compare both short‐ and long‐term clinical outcomes with younger patients. As suspected, we found an association between octogenarian patients and increased procedural complications, in‐hospital major adverse cardiac events, and long‐term all‐cause mortality, which confirms the findings of several recent studies.^2^, ^7^, ^19^, ^23^ Similarly, our study confirms that the annual proportion and absolute numbers of octogenarians gradually increased over the study period, making this an increasingly important patient group.^7^, ^8^ Our data, from one of the largest registries of PPCI cases in octogenarians collected over 7 years, demonstrate that the proportion of PPCI procedures for octogenarians increased annually to 10.5% in 2011, reflecting increasing numbers and experience of PPCI for octogenarians with STEMI.

In addition to these findings, we made several interesting observations. First, despite an increase in the mean age and the complexity of octogenarian patients undergoing PPCI, including more diabetes mellitus, previous MI and revascularization, and greater likelihood of presenting with cardiogenic shock, long‐term all‐cause mortality remained the same over time. This may be attributable to an improved PPCI technique in view of our findings of increased radial access, improved procedural success rates, and lower bleeding rates, although we are unable to comment on causality. This is consistent with the finding of Di Bari and colleagues that the application of PCI for acute coronary syndrome is associated with greater long‐term survival in patients aged >75 years with higher background risk, despite being applied less frequently in higher risk patients.^15^ In this regard, we hypothesize that the deleterious increasing age of the octogenarian cohort is offset by improved outcomes in higher risk patients, resulting in overall static long‐term all‐cause mortality. This may be a result of the observed increased application of evidence‐based interventions, including radial access and DESs, both of which may contribute to our finding of increased procedural success in more complex patients with STEMI over time.

Second, in‐hospital mortality of 7.7% for this octogenarian cohort versus 2.4% for younger patients was both reasonable and comparable to recently published studies in similar cohorts.^2^, ^19^ Nevertheless, our data are typical in demonstrating higher rates of cardiovascular risk factors, vascular disease in other distributions, worse left ventricular systolic function, and more cardiogenic shock, which is a particularly stronger predictor of mortality in elderly compared with younger patients, both in this study and others.^19^, ^24^, ^25^ Consequently, octogenarian patients undergoing PPCI for STEMI in this cohort had significantly higher 3‐year mortality than patients aged <80 years (51.6% vs 12.8%). This is similar to the limited number of studies to date that have examined long‐term outcomes after PPCI in octogenarians. Kvakkestad and colleagues, for example, found 58% 3‐year survival among octogenarians undergoing intervention for STEMI.^7^, ^8^, ^19^

Reasons for worse outcome after PPCI in elderly patients are likely to be multifactorial, including increased comorbidities, longer call‐to‐balloon times resulting in larger infarcts, reduced cardiovascular reserve, increased acute MI sequelae and more iatrogenic complications.^3^ Nearly all studies have shown, for example, increased access‐related complications, including bleeding events, in elderly patients^6^, ^26^ as well as higher risk of in‐hospital stroke.^17^, ^27^ Elderly patients are also more susceptible to contrast‐induced nephropathy after PCI on account of more complex lesions that may mandate increased contrast use^28^ and worse preexisting renal function.^29^

Third, the use of radial access, DES, GP IIb/IIIa inhibitors, and thrombectomy catheters was significantly less in the elderly cohort compared with the younger group, although both GP IIb/IIIa and thrombectomy use decreased over the study period in the whole cohort; this decrease may be explained by the publication of new trial data.^30^ Comorbidities may account for this disparity, including, for example, increased anemia, bleeding diatheses, and vascular disease; however, it is also possible that such interventions are underused in elderly patients because of concerns about increased risk and lack of available evidence. Although the evidence for thrombectomy and GP IIb/IIIa inhibition in STEMI is mixed,^30^, ^31^, ^32^, ^33^, ^34^, ^35^, ^36^ most studies investigating these interventions, as well as radial access and DES use, have excluded very elderly populations. After multivariate analysis of mortality following PPCI in the octogenarian cohort, the present study showed that, despite lower use of these therapies, radial access, GP IIb/IIIa inhibition, and DES use were associated with improved all‐cause mortality.

Only a small number of studies have examined the impact of procedural factors on outcome in elderly patients. A small observational study comparing octogenarian and younger patients receiving DESs for acute MI, unsurprisingly, found more cardiac death in the octogenarian cohort^37^; however, none of this excess mortality was due to stent thrombosis. Others have described a decline in DES use for all PCI in elderly patients, which interestingly did not appear to affect overall mortality, bleeding, or revascularization.^38^ Regarding access route, a study comparing radial and femoral access in octogenarians undergoing primary or rescue PCI demonstrated significantly higher bleeding complications in the femoral group and a higher conversion rate in the radial group, implying that radial access is feasible and safe in this cohort.^39^ Radial access may be preferable in elderly patients because they have significantly higher baseline risk of arterial access complications.^40^ It also facilitates early ambulation, which is particularly important for these patients who are at increased risk of venous thromboembolism and hospital‐acquired infections. Our observation that radial access is associated with improved outcome among octogenarians undergoing PPCI for STEMI may be confounded by case selection (whereby patients at highest risk preferentially receive femoral access); however, we previously described that patients with high baseline risk may, in fact, benefit most from radial access.^41^

Fourth, our study confirms that bleeding is an independent predictor of mortality in octogenarian and younger cohorts, which has been demonstrated in numerous randomized and observational studies.^42^ This result has been attributed to the discontinuation of antithrombotic medication, hypoperfusion, anemia, platelet activation, and/or the adverse effects of transfusion.^42^ Furthermore, we found that age is an independent predictor of bleeding.^43^, ^44^, ^45^ Interestingly, variables that were independently associated with bleeding complications were alike in both octogenarian and younger cohorts.

Finally, the present study identified longer call‐to‐balloon times despite similar door‐to‐balloon times and a lower rate of interhospital transfer among elderly patients. It is recognized that elderly patients often do not undergo PPCI for reasons including atypical or “silent” presentation, increased prevalence of left bundle‐branch block or residual ST‐segment elevation from old infarcts, reduced reporting of chest pain, and increased incidence of heart failure on presentation.^3^, ^46^ Furthermore, confusion may be the presenting feature in up to 20% of patients aged >85 years with acute MI, complicating diagnosis and management.^3^ The greater number of female octogenarians may exacerbate these trends.^47^ Our data may reflect reluctance to transfer elderly patients to tertiary units for PPCI for these reasons and possible concerns about care needs and quality of life or uncertainty or misconceptions about optimal care for the elderly.

### Strengths and Limitations of This Study

The strength of this study is that it includes patients from 8 different centers in a large metropolitan city with a diverse ethnic and social makeup. Our cohort includes large numbers of patients who either were brought directly to a PPCI center or, alternatively, were transferred after assessment in a non–PCI‐capable hospital. The study included patients with cardiogenic shock, previous bypass surgery, and other comorbidities and thus is representative of the broad variety of patients encountered in day‐to‐day clinical practice. Although inclusion of such patients may result in selection bias, the baseline characteristics were similar, and any differences were adjusted for in the multivariate analyses. Mortality tracking in England is particularly robust based on official UK Office of National Statistics data, and thus our mortality end point is reliable. The multivariate analyses highlight the quality of the data with well‐recognized predictors of mortality associated with adverse outcome in our data set.

Nevertheless, our study has limitations. First, our study applies only to patients with STEMI who received PPCI and not to octogenarians undergoing PCI for other indications. In addition, this study has all of the limitations of a registry and all of the potential bias and residual confounding associated with nonrandomized studies; therefore, the observed effect may be caused by unrecorded variables. We were not able to adjust for missing variables and those not collected by the database. In addition, we cannot exclude the possibility of underreporting of complications, although the tracking of mortality is robust, and we included only the 88% of patients who had definitive mortality data in our study cohort. It is possible that some patients in the elderly group died prior to getting to the PCI center, and that could have counterbalanced the longer treatment times. Finally, although only a patient's first STEMI was included, repeat events at a different institution could not be identified after electronic encryption and merging of data sets from each institution; however, the numbers are likely to be small, and this should not have affected the primary mortality end point.

## Conclusions

In this large registry, octogenarians undergoing PPCI had a higher rate of complications and mortality compared with a younger population. Over time, octogenarians undergoing PPCI increased in number, age, and complexity. Nevertheless, mortality rates were static, which may be attributable to improved PPCI techniques, although further, randomized studies are necessary. Despite the advanced age of the study population, we suggest that the favorable short‐term outcomes described—in the absence of chronic kidney disease, left ventricular failure, and cardiogenic shock—justify consideration of PPCI for octogenarians with STEMI.

## Sources of Funding

Dr Daniel Bromage is an MRC Clinical Research Training Fellow supported by grant MR/L002043/1.

## Disclosures

None.
